# Influence of configuration and anchor in ligamentous augmentation to prevent proximal junctional kyphosis: A finite element study

**DOI:** 10.3389/fbioe.2022.1014487

**Published:** 2022-11-04

**Authors:** Wei Liu, Lei Zang, Nan Kang, Lihui Yang, Likun An, Wenyi Zhu, Yong Hai

**Affiliations:** Department of Orthopedic Surgery, Beijing Chao-Yang Hospital, Capital Medical University, Beijing, China

**Keywords:** adult spinal deformity, finite element, ligaments, proximal junctional kyphosis, tether

## Abstract

Ligament augmentation has been applied during spinal surgery to prevent proximal junctional kyphosis (PJK), but the configuration and distal anchor strategies are diverse and inconsistent. The biomechanics of different ligament augmentation strategies are, therefore, unclear. We aimed to create a finite element model of the spine for segments T6–S1. Model Intact was the native form, and Model IF was instrumented with a pedicle screw from segments T10 to S1. The remaining models were based on Model IF, with ligament augmentation configurations as common (CM), chained (CH), common and chained (CHM); and distal anchors to the spinous process (SP), crosslink (CL), and pedicle screw (PS), creating SP-CH, PS-CHM, PS-CH, PS-CM, CL-CHM, CL-CH, and CL-CM models. The range of motion (ROM) and maximum stress on the intervertebral disc (IVD), PS, and interspinous and supraspinous ligaments (ISL/SSL) was measured. In the PS-CH model, the ROM for segments T9–T10 was 73% (of Model Intact). In the CL-CHM, CL-CH, CL-CM, PS-CM, and PS-CHM models, the ROM was 8%, 17%, 7%, 13%, and 30%, respectively. The PS-CH method had the highest maximum stress on IVD and ISL/SSL, at 80% and 72%, respectively. The crosslink was more preferable as the distal anchor. In the uppermost instrumented vertebrae (UIV) + 1/UIV segment, the CM was the most effective configuration. The PS-CH model had the highest flexion load on the UIV + 1/UIV segment and the CL-CM model provided the greatest reduction. The CL-CM model should be verified in a clinical trial. The influence of configuration and anchor in ligament augmentation is important for the choice of surgical strategy and improvement of technique.

## 1 Introduction

Adult spinal deformity (ASD) is a common spine disorder, especially in the elderly population (Schwab et al., 2005), that can lead to chronic pain, functional impairment, and great physical and psychological distress in patients (Pellise et al., 2015). Some patients require surgical intervention, and long-segment instrumented spinal fusion remains the most commonly used method to treat this disorder. PJK is a common complication after long-segment fixation surgery of the spine. It is generally defined as 1) a proximal junction sagittal Cobb angle greater than or equal to 10°, and 2) a proximal junction sagittal Cobb angle at least 10° greater than the preoperative measurement (Glattes et al., 2005). The incidence of PJK ranges from 20% to 39% (Glattes et al., 2005; Kim et al., 2008; Yagi et al., 2011), with 59% of PJK instances occurring within 8 weeks after surgery, 35% occurring between 8 weeks and 2 years after surgery, and 6% occurring more than 2 years after surgery (Kim et al., 2008). PJK can be further aggravated, leading to proximal junctional failure (PJF), which causes neurological impairment and mostly requires revision surgery (Hostin et al., 2013). The prevention of PJK is, therefore, of great importance, and the current primary intraoperative prevention strategy is prophylactic vertebral augmentation (Hart et al., 2008), hook (Helgeson et al., 2010), or ligament augmentation (Safaee et al., 2017).

Since the posterior ligament complex plays an important role in stabilizing the spine (Heuer et al., 2007; Anderson et al., 2009), the technique of ligament augmentation chosen may preserve the intervertebral joint and reduce the occurrence of PJK. Most trials using ligament augmentation in the clinic to reduce PJK occurrence have resulted in good clinical outcomes (Safaee et al., 2018a; Safaee et al., 2018b; Buell et al., 2019b; Buell et al., 2019c; Rodriguez-Fontan et al., 2020; Rabinovich et al., 2021; Rodnoi et al., 2021) and have provided an improved cost-benefit ratio than when patients did not undergo ligament augmentation (Safaee et al., 2018a). However, other studies have concluded that ligament augmentation does not reduce the incidence of PJK (Iyer et al., 2020; Alluri et al., 2021). One possible reason for the contradictions in the above reports is the use of different ligament augmentation strategies, including different ligament augmentation configurations and anchoring points.

Different ligament augmentation strategies may affect the biomechanics of adjacent segments of the UIV, and the literature surrounding this remains scarce (Buell et al., 2019a; Mar et al., 2019a). Buell et al. (2019a) agreed that only the level of the augmentation segment affects the result, not the configuration and anchor; but Mar et al. (2019a) concluded that the tether configuration significantly alters segmental biomechanics. Thus, we aimed to develop a finite element model with augmentation at UIV + 2 and UIV + 1, using anchors to the spinous process (SP), crosslink (CL), or pedicle screw (PS) and three configuration methods [chained (CH), common (CM), and common-chained (CHM)] to investigate the anchor point and configuration effect of the adjacent segment of the UIV.

## 2 Materials and methods

### 2.1 Geometry model

All activities were approved by the Ethics Committee of Beijing, Chaoyang Hospital, Capital Medical University (IRB number 2018-4-10-3), and the participant was required to sign an informed consent form. Computed tomography scan data from a healthy 47-year-old male, using a 512 × 512-pixel matrix at 1-mm intervals, were used to construct a three-dimensional (3D) model of the human T6–S1 segment. The participant’s CT images were imported into Mimics software (Mimics 10.0; Materialise Technologies, Leuven, Belgium) and a 3D geometric model of the vertebral body and intervertebral discs was built.

#### 2.1.1 3D geometric model of the vertebral body

The vertebral body was segmented by the difference in gray values between the bone and the surrounding tissues. The segmented vertebral body image data were then reconstructed, to generate a T6–S1 3D geometric model. Finally, the 3D vertebral body model was smoothed.

#### 2.1.2 3D geometric model of the intervertebral disc

The segmented two-dimensional (2D) image data of the intervertebral discs, distinguished by the nucleus pulposus and annulus fibrosus, were reconstructed to generate a 3D geometric model of the T6–S1 intervertebral discs. The model was then smoothed.

### 2.2 Finite element model

HyperMesh version 11.0 (Altair Engineering, Inc., Troy, MI) was used to mesh the vertebral body and intervertebral discs. The insertion point of each ligament was connected in HyperMesh according to known anatomical features, to establish long ligaments (anterior longitudinal, posterior longitudinal, supraspinous, and interspinous) and short ligaments (ligamentum flavum, intertransverse, and capsular). The meshed vertebral body, ligaments, and intervertebral discs were then imported into Abaqus version 6.11 (Dassault Systèmes Simulia Corp., Providence, RI) for simulation. The spine material properties were derived from published studies (Sairyo et al., 2006; Fan et al., 2010; Liu et al., 2011) (Table 1).

**TABLE 1:** Summary of material properties in the finite element model.

Position	Young’s modulus (MPa)	Poisson’s ratio	Cross-section (mm)	Reference
Cortical bone	12,000	0.3	—	Fan et al. (2010)
Cancellous bone	100	0.2	—	Fan et al. (2010)
Endplate	3,000	0.25	—	Sairyo et al. (2006)
Anterior longitudinal ligament	15	—	40	Fan et al. (2010)
Posterior longitudinal ligament	10	—	20	Fan et al. (2010)
Supraspinous ligament	8	—	30	Fan et al. (2010)
Interspinous ligament	10	—	40	Fan et al. (2010)
Ligamentum flavum	15	—	40	Fan et al. (2010)
Intertransverse ligament	10	—	1.8	Fan et al. (2010)
Capsular	7.5	—	30	Fan et al. (2010)
Nucleus pulposus	1.0	0.499	—	
Annulus fibrosus	4.2	0.45	—	Sairyo et al. (2006)
Pedicle screw and rod (Ti)	1,10,000	0.28	—	Fan et al. (2010)
Augmentation ligament (PET)	1,500	0.4	—	Liu et al. (2011)

### 2.3 Simulated differences with ligament augmentation

**FIGURE 1:**
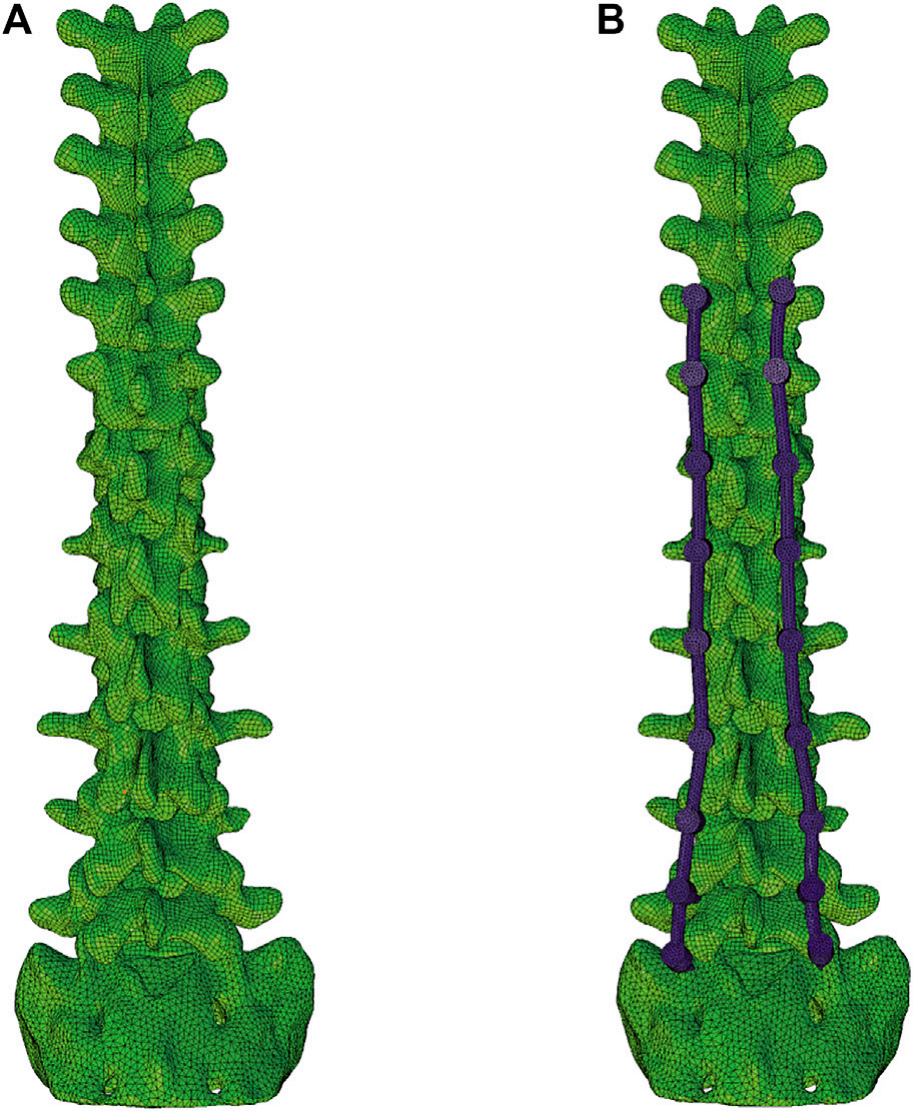
Finite element models. Model intact **(A)** was the native form, and model IF **(B)** was instrumented with a pedicle screw from segments T10 to S1.

**FIGURE 2:**
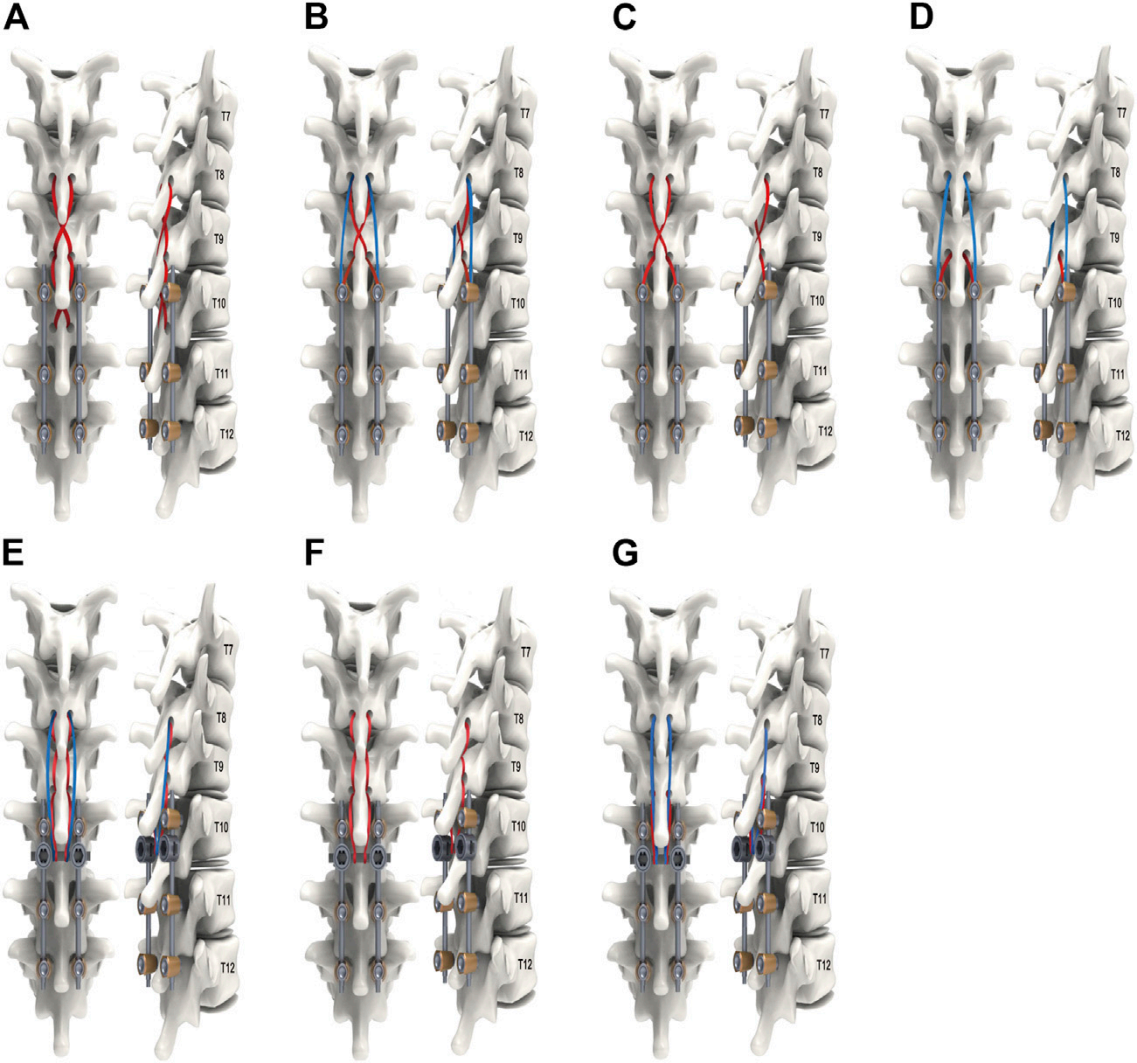
The remaining models, based on model IF with ligament augmentation configuration of common (CM), chained (CH), and common and chained (CHM) models; distally anchored to the pedicle screw (PS), crosslink (CL), or the spinous process (SP): **(A)** Model SP-CH; **(B)** Model PS-CHM; **(C)** Model PS-CH; **(D)** Model PS-CM; **(E)** Model CL-CHM; **(F)** Model CL-CH; and **(G)** Model CL-CM.

The following nine models were considered. Model Intact was the finite element model without surgery. Model IF reflected a fixed arrangement with a long-segment PS from T10 to S1 segments (Figure 1). All remaining models were based on Model IF with ligament augmentation. The ligament augmentation configurations were defined as: 1) loop configuration, meaning that the proximal fixation point was directly linked to the distal anchor without a tether to the adjacent segment; 2) weave configuration, the proximal fixation point was tethered to the adjacent segment and then linked to the distal anchor (Buell et al., 2019a); 3) CM, the UIV + 2/UIV loop was combined with the UIV + 1/UIV loop; 4) CH, the UIV + 1/UIV loop was combined with the UIV + 2/UIV + 1 weave (Mar et al., 2019a); 5) CHM, included the UIV + 2/UIV loop, UIV + 1/UIV loop, and UIV + 2/UIV + 1 weave. The distal anchor points were the SP, PS, or CL. Models SP-CH, PS-CH, PS-CM, PS-CHM, CL-CH, CL-CM, and CL-CHM are shown in Figure 2.

On the upper endplate of the T6 vertebra, a 5 N moment was used to simulate forward bending (Liu et al., 2011). Tie constraints were applied to simulate the connected relationship between the vertebra and disc, between the vertebral body and ligament, between the vertebra and screws, and between the vertebral body and the augmentation ligament, so that these connections were kept constant during movement under stress. The lower sacrum was constrained to be fixed. ROM and maximum stress on the IVD, PS, and of the ISL/SSL were measured.

Validation of the model was performed by comparing the model ROM to results in previous studies (Morita et al., 2014; Couvertier et al., 2017; Ignasiak et al., 2017). The ROM of the T8–T10 segments in the Model Intact was in agreement with that in the published experimental results.

## 3 Results

### 3.1 Range of motion

**FIGURE 3:**
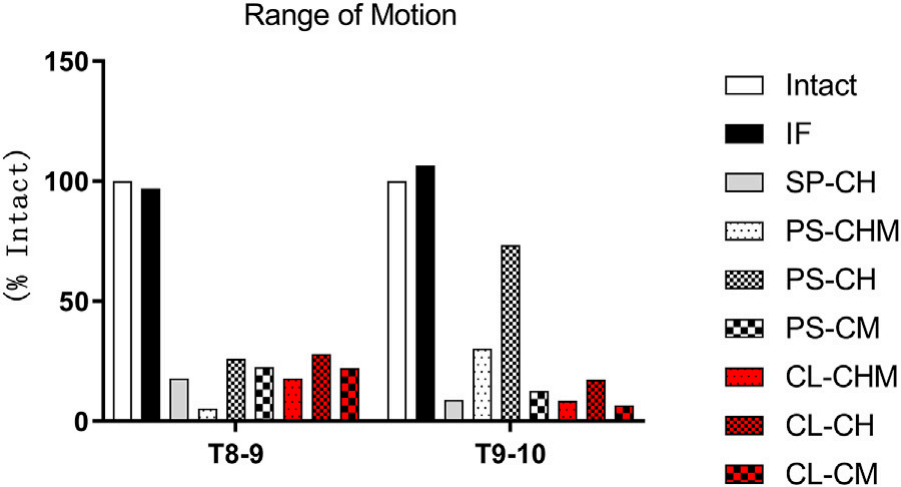
Range of motion (% ROM in model intact), measured at the T8–9 and T9–10 segments for model intact and IF, SP-CH, PS-CHM, PS-CH, PS-CM, CL-CHM, CL-CH, and CL-CM models.

Compared to Model Intact, in Model IF, the ROM was 97% intact for segments T8–9 and 107% intact for segments T9–10. For segments T8–9, the ROM in each ligament augmentation group was significantly reduced, with the lowest value for PS-CHM being 5% and the highest value for CL-CH being 28%. For T9–10 segments, PS-CHM and PS-CH models had ROMs of 30% and 73%, respectively, while CL-CHM and CL-CH models had ROMs of 8% and 17%, respectively. The ROM was 13% for PS-CM and 7% for CL-CM models (Figure 3).

### 3.2 Maximum stress on intervertebral disc

**FIGURE 4:**
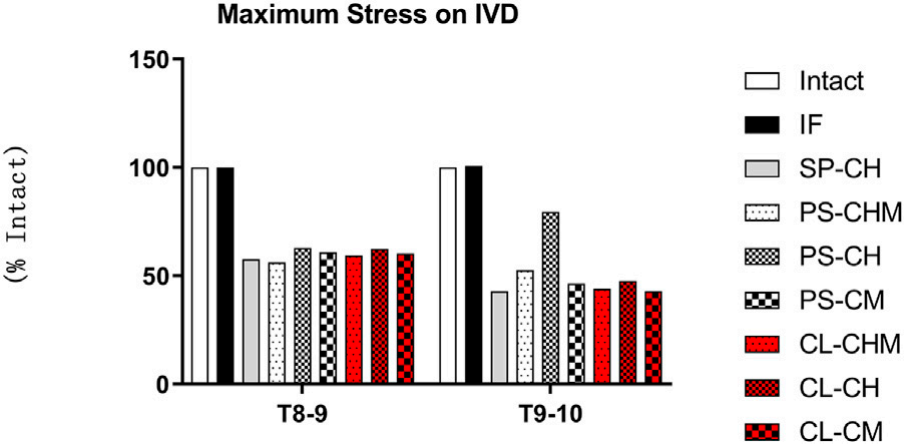
Maximum stress of the intervertebral disc (% of maximum stress of the intervertebral disc in model intact), measured at T8–9 and T9–10 segments for model intact, and IF, SP-CH, PS-CHM, PS-CH, PS-CM, CL-CHM, CL-CH, and CL-CM models.

Compared with Model Intact, the maximum stress of the intervertebral disc in Model IF was 100% intact at segments T8–9 and 101% intact at segments T9–10. At the T8–9 segments of the SP-CH, PS-CHM, PS-CH, PS-CM, CL-CHM, CL-CH, and CL-CM models, the maximum stress of the intervertebral disc was reduced to 58%, 57%, 63%, 61%, 59%, 63%, and 60%, respectively. For segments T9–10, the lowest maximal stress was observed for SP-CH and CL-CM models at 43% of that of Model Intact, and the highest was in model PS-CH at 80%. PS-CM, PS-CHM, CL-CH, and CL-CHM models had maximal intervertebral disc stresses of 46%, 53%, 48%, and 44%, respectively (Figure 4).

### 3.3 Maximum stress of pedicle screws

**FIGURE 5:**
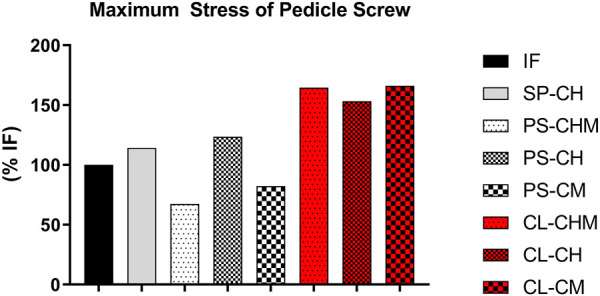
Maximum stress of the pedicle screw (% of maximum stress of pedicle screw in model IF), measured at the segment T10 pedicle screw for IF, SP-CH, PS-CHM, PS-CH, PS-CM, CL-CHM, CL-CH, and CL-CM models.

Models PS-CHM and PS-CM reduced the maximum stress on the T10 pedicle screw by 68% and 82%, respectively. The PS-CH model did not reduce the maximum stress on the T10 pedicle screw. The maximal stress at the PS increased by 165%, 153%, and 166% for CL-CH, CL-CHM, and CL-CM models, respectively (Figure 5).

### 3.4 Maximum stress of interspinous and supraspinous ligaments

**FIGURE 6:**
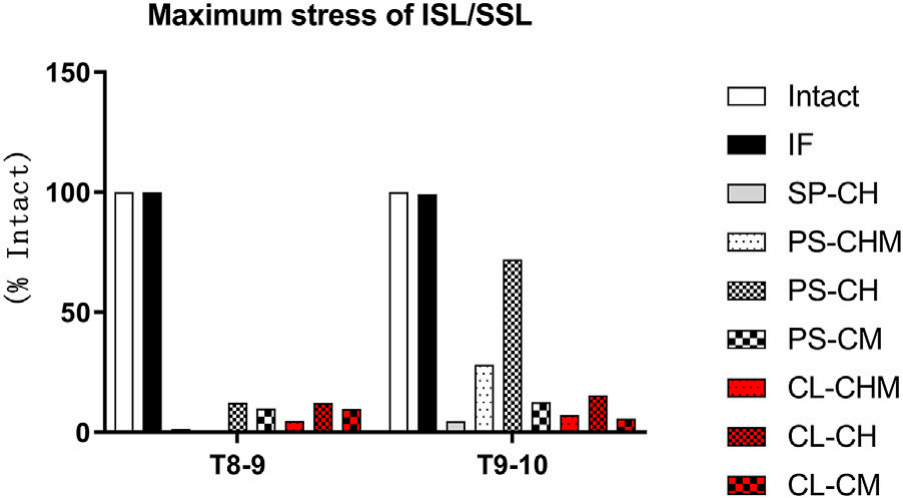
Maximum stress of the ISL/SSL (% of maximum stress of the ISL/SSL in model intact), measured at T8–9 and T9–10 segments for intact, IF, SP-CH, PS-CHM, PS-CH, PS-CM, CL-CHM, CL-CH, and CL-CM models.

Relative to Model Intact, no significant changes in the maximum stress of ISL/SSL were observed after pedicle screw fixation at segments T8–T9 or T9–T10. At segments T8–T9, models SP-CH and PS-CHM stresses were significantly reduced to 1% of that of Model Intact and 0% of Model Intact, respectively, while that of models PS-CH, PS-CM, CL-CHM, CL-CH, and CL-CM were 12%, 10%, 5%, 12%, and 10%, respectively. In the T9–10 segments, the stresses of models SP-CH, CL-CHM, and CL-CM decreased significantly, to 5%, 7%, and 6%, respectively. The maximum stresses in models PS-CM and CL-CH were 12% and 15%, respectively, while the maximum stresses in models PS-CHM and PS-CH were 28% and 72%, respectively (Figure 6).

## 4 Discussion

Although the technique of ligament augmentation has been applied in clinical practice, the strategy varies widely in terms of anchoring point and fixation configuration, with no standard or ideal method, which has led to variable clinical outcomes. Rodriguez-Fontan et al. (2020) applied Mersilene tape stabilization in the thoracic spine to encircle the UIV + 1 and UIV + 2 spinous processes, which were then fixed to the bilateral connecting rods or crosslink. This approach is comparable to our PS-CH or CL-CH models. Only the fixation of the UIV + 1 SP was enhanced in the lumbar spine. This indicates that the risk of PJK can be reduced with this method. Notably, Buell et al. (2019b) used Mersilene tape in a clinical setting through perforation of the base of the UIV + 1 SP, and fixed it distally to either the UIV-1 SP or the crosslink in a loop configuration, and found that this tether technique could reduce the risk of PJK. In particular, this modality of fixation to the crosslink was statistically significant compared with that of the matched control group. Rabinovich et al. (2021) reinforced the UIV + 2 or UIV + 1 segment with a loop configuration, and concluded that the tethered distal anchor to the crosslink could significantly reduce the incidence of PJK. Rodnoi et al. (2021) used a similar tether technique to lower the risk of PJK. Safaee et al. (2018b) used sublaminar cables to encircle the SPes of UIV + 1, UIV, and UIV-1 and connected them to the bilateral rods; as a result, the incidence of PJK was significantly reduced. Iyer et al. (2020) used loop fixation to attach the UIV and UIV + 1 spines anchored to bilateral rods in the clinic, to enhance the UIV + 1/UIV segments, but this did not significantly decrease the incidence of PJK after 1 year compared to controls. Alluri et al. (2021) used a semitendinosus tendon to augment UIV + 1, UIV, UIV-1, and UIV-2 with attachments to crosslinks. This technique led to a significant reduction in the occurrence of PJF and an improved function and prognosis but did not significantly reduce the occurrence of PJK. These differences in clinical outcomes imply that different anchoring points and fixation configurations have the potential to influence the stiffness of the ligament augmentation.

Current biomechanical results show that augmentation of only UIV + 1/UIV segment leads to poor biomechanical effects. Bess et al. (2017) conducted a finite element biomechanical study of ligament augmentation for the prevention of PJK, to show that increasing the number of ligament augmentation segments could decrease the ROM, intradiscal pressure, PS force, and posterior ligament complex force. Thus, ligament augmentation may produce a gradual transition between fused and non-fused segments that can reduce the biomechanical risk of PJK. Kim et al. (2019) created a cadaveric model containing a native spine, a fused spine, a fused spine with UIV + 1/UIV looped by a tether, and a fused spine with a cut off at the posterior ligament complex. The fused spines with UIV + 1/UIV looped by tethers failed to exhibit reduced segmental ROM and flexion load.

Our study considered ligament augmentation of segments at two levels, UIV + 1 and UIV + 2, anchored distally to the crosslink, SPes, or PSs, and combined distal fixation points and configurations to compare their mechanical effects. In the UIV + 2/UIV + 1 segment, the CHM configuration was the most effective method, and the CM was better than the CH. In the UIV + 1/UIV segment, the most effective configuration was CM; CHM was better than the CH. Compared with the distal anchor in the UIV + 1/UIV segment, the crosslink was superior to the PS; in the UIV + 2/UIV + 1 segment, the crosslink was no different to the PS. Considering the distal anchor and configuration in UIV + 1/UIV, the PL-CH was the least able to reduce the ROM and maximum stress on IVD. The CL-CM had the greatest biomechanical effect. Our result shows that the distal anchor to the crosslink was better than to the SP and PS. This is consistent with the results of studies by Buell et al. (2019b), Rabinovich et al. (2021), and Rodnoi et al. (2021). Moreover, the tether tape could be tensioned by compressing the crosslink. The distal anchor to the SP also had some disadvantages. With a high preload of the tether or low bone mineral density of SP, the risk to the SP bone failure is high (Mar et al., 2019b). Although the CL-CM method was the most effective in reducing the load of the UIV + 1/UIV segment in our study, clinical evidence remains scarce. Clinical studies regarding reinforcement of two segments have been conducted by Rodriguez-Fontan et al. (2020) (PS-CH/CL-CH method) and Rabinovich et al., 2021 (loop method). The advantages of the CL-CM method warrant future research.

The biomechanical research results of ligament augmentation of the UIV + 2/UIV + 1 and UIV + 1/UIV segments are shown in Table 2. Mar et al. (2019a) had cadaveric experimental results similar to ours, and their CM and CH models were equivalent to our CL-CM and CL-CH models. The CH and CM method reduced the IDP, ROM, and maximum tether force in the UIV/UIV + 1 segment but made little difference in the UIV + 1/UIV + 2 segment. In the UIV + 1/UIV segment, the CM method was superior to the CH method. In our study, the CL-CM method provided more power segment biomechanical effect than the CL-CH model, not only in the UIV + 1/UIV segment but also in the UIV + 2/UIV + 1 segment. The difference in these results may be related to the thoracic region studied. We chose to focus on the lower thoracic area, while the cadaver test was conducted on the upper thoracic region. Additionally, a preload of 22 N was applied in the cadaver test, so that more of the load was shared by the tether. In the study by Zhao et al. (2022) the TE-T6–T9 model was similar to our SP-CH model. In the study by Bess et al. (2017), the TE-UIV+2 model was similar to our PS-CM model. Bess et al. (2017) and Zhao et al. (2022) did not compare the different configurations and anchors, but implied that two-segment ligament augmentation compared to one-segment could produce more gradual transition between fused and non-fused segments.

**TABLE 2:** List of biomechanical research tests for ligament augments in UIV + 2/UIV + 1 and UIV + 1/UIV segments compared with distal anchor, configuration, and parameters.

Reference	Distal anchor	Configuration	Segment	ROM (%)	ROM (degrees)	IDP (%)	Maximum stress on IVD (Mpa)	ISL/SSL force (%)	Pedicle screw force (N)	Maximum tether force (N)	Maximum stress in vertebrae (N)	Maximum stresses of pedicle screws (Mpa)	Maximum stress of ISL/SSL (Mpa)
Bess et al. (2017)	Pedicle screw	CM	UIV + 1/UIV	51		69		53	23.6				
			UIV + 2/UIV + 1	69		81		66	5				
Mar et al. (2019a)	Crosslink	CM	UIV + 1/UIV	57		56				16			
			UIV + 2/UIV + 1	91		79				53			
		CH	UIV + 1/UIV	83		82				34			
			UIV + 2/UIV + 1	94		81				55			
Buell et al. (2019a)	Spinous process or rod	Loop	UIV + 1/UIV	22		45		15	19				
			UIV + 2/UIV + 1	26		5		26					
		Weave (CH)	UIV + 1/UIV	24		47		15	17				
			UIV + 2/UIV + 1	30		53		30					
Zhao et al. (2022)	Spinous	CH	UIV + 1/UIV	26			36				52.08	44.07	
			UIV + 2/UIV + 1	30			36.8				50.12		
Present study	Spinous	CH	UIV + 1/UIV	8.9	0.086		0.216					30.63	0.048
			UIV + 2/UIV + 1	17.8	0.133		0.286						0.017
	Pedicle Screw	CHM	UIV + 1/UIV	30.3	0.292		0.266					18.06	0.295
			UIV + 2/UIV + 1	5.3	0.04		0.279						0
		CH	UIV + 1/UIV	73.3	0.706		0.401					33.1	0.752
			UIV + 2/UIV + 1	26.1	0.195		0.312						0.145
		CM	UIV + 1/UIV	12.7	0.123		0.234					22.1	0.132
			UIV + 2/UIV + 1	22.5	0.168		0.302						0.118
	Crosslink	CHM	UIV + 1/UIV	8.5	0.082		0.222					44.1	0.075
			UIV + 2/UIV + 1	17.8	0.133		0.295						0.055
		CH	UIV + 1/UIV	17.3	0.167		0.24					41.1	0.161
			UIV + 2/UIV + 1	28	0.209		0.31						0.144
		CM	UIV + 1/UIV	6.7	0.066		0.216					44.5	0.059
			UIV + 2/UIV + 1	22	0.164		0.299						0.114

The conclusion from the study by Buell et al. (2019a) differ from ours. While the UIV + 2 weave tethers were similar to our SP-CH and PS-CH models, they found that the UIV + 2 loop or weave tether was the best mode of augmentation, whereas the distal fixation point and loop or weave configuration had no apparent effect on the results. However, the method of anchoring to the crosslink was not considered, nor was the configuration of CM and CHM; but the crosslink was the distal anchor which proved to be effective (Buell et al., 2019b; Rabinovich et al., 2021; Rodnoi et al., 2021). The UIV + 2 loop method only tethered with the UIV + 2 SP without the tether to the UIV + 1 SP. The load on the UIV + 1/UIV segment could not be effectively shared with the tether, which may have caused the indifference with the UIV + 2 loop and UIV + 2 weave.

Our research has some limitations. Firstly, no effect of preload on the tether was considered in the conclusion, and manual or device preload is used in clinic. Buell et al. (2019b) reported that the crosslink was retracted to full tension to create a preload on the polyethylene tape, and the results were statistically significant compared to those of the control group. The results of the finite element model by Buell et al. (2019a) showed that ROM and IDP could be reduced within a 100 N preload. Mar et al. (2019b) also concluded from cadaveric specimens that an increased preload on the tape reduced IDP and ROM. Secondly, our IVD finite element model was relatively simple, in which we assumed that the nucleus pulposus was simulated as an incompressible fluid and the nonlinear annulus fibrosus was simulated by hyper-elastic Mooney–Rivlin formulation. A more precise model should be considered in the future, in which the nucleus pulposus and annulus fibrosus are modeled as biphasic materials composed of a solid matrix surround in a fluid phase (Elmasry et al., 2017; Elmasry et al., 2018). Thirdly, we only simulated the lower thoracic spine and did not simulate upper thoracic surgery. Upper and lower thoracic surgery have clinically different outcomes, with higher pseudoarthrosis in the upper thoracic spine and a higher incidence of PJK in the lower thoracic spine (O’Shaughnessy et al., 2012). Finally, resection of the ribs has a significant impact on the stiffness of the thoracic spine (Little and Adam, 2011; Anderson et al., 2018). Furthermore, preoperative lower muscularity can lead to PJK (Hyun et al., 2016), and we did not consider the effect of the ribs and muscles on the results. Despite the above mentioned limitations, our study is one of relatively few articles that consider the biomechanical effects of the configuration of ligament augmentation and the distal anchoring position on the prevention of PJK. The influence of configuration and anchor in ligament augmentation is important for the choice of surgical strategy and improvement of technique.

## 5 Conclusion

Different anchor points, configurations, and combinations of both affect the biomechanics of the adjacent segment to UIV. The crosslink is more preferable than the PS or SP compared to the distal anchor. In the UIV + 1/UIV segment, the CM configuration was the most effective. The PS-CH model had the highest flexion load on the UIV + 1/UIV segment and the CL-CM model provided the greatest reduction. The CL-CM has good biomechanic effects, so it has clinical potential to better predict functional outcomes. The CL-CM model should be verified in a clinical trial.

## Data Availability

The raw data supporting the conclusion of this article will be made available by the authors, without undue reservation.

## Abbreviations

ASD, adult spinal deformity; CH, chained; CHM, common and chained; CL, crosslink; CM, common; IDP, intradiscal pressure; ISL/SSL, interspinous and supraspinous ligaments; IVD, intervertebral disc; PET, polyethylene terephthalate; PJK, proximal junctional kyphosis; PS, pedicle screw; ROM, range of motion; SP, spinous process; Ti, titanium; UIV, uppermost instrumented vertebrae.
